# Knowledge, attitudes, and practices regarding fertility preservation among women of childbearing age in southern China: a cross-sectional study

**DOI:** 10.3389/fpubh.2025.1612784

**Published:** 2025-10-03

**Authors:** Yan Zhang, Juan An, Fanglan Hua, Yanhong Wei, Fanglian Shu

**Affiliations:** ^1^Department of Gynecology and Obstetrics, The People’s Hospital of Suzhou New District, Suzhou, China; ^2^Department of Ophthalmology and Otorhinolaryngology, The People’s Hospital of Suzhou New District, Suzhou, China

**Keywords:** fertility preservation, health belief model, reproductive health, women’s health, health behaviour, cross-sectional study

## Abstract

**Objective:**

This study aimed to investigate the knowledge, attitudes and practices regarding fertility preservation among women of childbearing age and to analyse the factors influencing these behaviours.

**Methods:**

A cross-sectional survey of 294 women of childbearing age (18–49 years) was conducted in urban healthcare settings between January 2024 and March 2024 using convenience sampling; it assessed fertility preservation behaviours and health beliefs through a structured questionnaire and multiple regression analysis.

**Results:**

Among the participants, 62.93% reported prior awareness of fertility-related concepts. Healthcare workers constituted the largest occupational group (45.24%), and 80.61% had a college education or above. High compliance was observed in personal hygiene (85.03%) and avoidance of smoking (71.77%), whereas only 31.97% regularly underwent fertility assessments. Health belief analysis revealed high levels of perceived susceptibility (87.41%) and perceived benefits (89.79%). Multiple regression analysis identified education level (*β* = 0.326, *p* < 0.001), healthcare occupation (*β* = 0.284, *p* < 0.001) and perceived benefits (*β* = 0.253, *p* < 0.001) as significant predictors of fertility preservation behaviours. Main barriers included time constraints (50.34%) and financial concerns (25.17%).

**Conclusion:**

Despite high awareness of the importance of fertility preservation, significant gaps exist between knowledge and practice. Education level, healthcare occupation and perceived benefits strongly influence protective behaviours. These findings suggest the interventions should not only enhance health education but also address structural obstacles, such as cost and accessibility, to bridge the gap between knowledge and action.

## 1 Introduction

As the global population shifts toward childbearing later in life and declining fertility rates (1), fertility preservation is increasingly recognised as an important reproductive health priority. Recent data indicate that more women are postponing childbearing for educational pursuits, career development or personal reasons; as a result, the average age of first pregnancy has risen markedly over the past few decades (2). This demographic shift, combined with various environmental and lifestyle factors, has made fertility preservation an increasingly important consideration for women of childbearing age (3).

The concept of fertility preservation encompasses a range of preventive behaviours and medical interventions designed to maintain or protect reproductive potential. These include lifestyle modifications, environmental exposure prevention and medical approaches (4). However, research suggests that awareness and implementation of fertility preservation strategies remain suboptimal among women of reproductive age, potentially impacting future reproductive outcomes (5). The health belief model (HBM) has proven particularly valuable in explaining these gaps; studies have demonstrated that women’s perceived susceptibility to fertility problems and their perceived benefits of protective behaviours largely predict the adoption of protective behaviours (6).

Although previous studies have identified socioeconomic status, education level, healthcare access and cultural beliefs as key determinants of fertility preservation behaviours (7), several barriers persist in implementing protective behaviours. These barriers include limited knowledge about fertility risks, misconceptions about fertility decline, inadequate healthcare resources and financial constraints (8). The COVID-19 pandemic has disrupted reproductive health services, thereby exacerbating these challenges and further highlighting the need for robust fertility preservation measures (9).

Emerging evidence highlights additional complexities, indicating that exposure to certain chemicals, radiation and other workplace hazards has a significant impact on reproductive health outcomes (10). Although lifestyle factors, such as nutrition, physical activity and stress management, have been shown to have an impact, they are not given enough attention in current fertility preservation paradigms (11). Furthermore, although healthcare providers are important promoters of fertility preservation awareness and practices, studies have shown that communication between healthcare providers and patients has been deficient, particularly in primary care settings (12, 13).

Therefore, current research on how specific health beliefs interact with demographic factors to form fertility preservation behaviours is limited; there have been insufficient investigations into practical barriers beyond economic constraints, and there is a lack of comprehensive assessments of knowledge and practice. Our study employs a multidimensional approach to investigate the knowledge, attitudes and practices regarding fertility preservation among women of childbearing age and to analyse the factors influencing these behaviours, addressing these limitations. Understanding these aspects is crucial for providing evidence for targeted interventions that can bridge the gap between knowledge and practice and optimise the outcomes of fertility preservation.

## 2 Methods

### 2.1 Study design and setting

A cross-sectional survey was conducted between January 2024 and March 2024 at multiple healthcare institutions in urban areas. This study employed a convenience sampling method and was approved by the institutional ethics committee (No. 2024-090); it was performed in line with the principles of the Declaration of Helsinki. All participants provided informed consent before participating in the study.

### 2.2 Participants

The study population comprised women of childbearing age (18–49 years) attending routine health check-ups or gynaecological clinics. Women who were unable to comprehend the questionnaire or had severe physical or mental conditions that may have affected their ability to participate were excluded from the study. A total of 294 eligible participants were ultimately enrolled.

### 2.3 Questionnaire design

Data collection was performed using a structured questionnaire that consisted of three main sections: (1) demographic information, including age, residence, education level, marital status, number of children, economic status, occupation and medical insurance coverage; (2) assessed fertility preservation behaviours through 24 items covering various aspects, such as dietary habits, exercise patterns, lifestyle choices and healthcare-seeking behaviours. Each item was rated on a five-point Likert scale ranging from ‘almost never’ (1 point) to ‘very often’ (5 points). The composite behaviour score was calculated by direct arithmetic summation of all 24 items, with higher scores indicating more protective behaviours; (3) evaluated health beliefs regarding fertility preservation using the HBM framework. This section included five dimensions – perceived susceptibility (6 items) assessed subjective perception of risk for reproductive health damage; perceived severity (6 items) evaluated the perceived impact of fertility problems on life and pregnancy outcomes; perceived benefits (6 items) measured beliefs about positive outcomes from protective behaviours; perceived barriers (8 items) identified obstacles to implementing protective behaviours; and self-efficacy (5 items) assessed confidence in overcoming barriers and maintaining long-term changes. All items were rated on a five-point Likert scale from ‘strongly disagree’ (1 point) to ‘strongly agree’ (5 points). Negatively worded items were reverse-coded before summation. For the regression analysis, each subscale score was standardised using a z-score transformation. Complete item lists and scoring methods are provided in Supplementary Table S1.

The questionnaire was developed based on an extensive literature review and expert consultation. Content validity was established through review by a panel of five experts in reproductive health and health education. A pilot study was conducted with 30 participants (not included in the final analysis) to assess the questionnaire’s clarity and feasibility. The Cronbach’s alpha coefficient for the overall questionnaire was 0.87, indicating good internal consistency.

### 2.4 Data collection

Trained research assistants distributed the questionnaires in private settings within the healthcare facilities. Participants completed the questionnaires independently, with assistance available if needed for clarification. Each questionnaire took approximately 15–20 min to complete. To ensure data quality, completed questionnaires were reviewed for completeness and accuracy before participants left the survey site.

### 2.5 Statistical analysis

Statistical analysis was performed using SPSS version 26.0 (IBM Corp., Armonk, NY, USA). Descriptive statistics were calculated for demographic characteristics and questionnaire responses. Continuous variables were presented as means and standard deviations, and categorical variables were expressed as frequencies and percentages. The relationships between demographic characteristics and fertility preservation behaviours were examined using chi-squared tests for categorical variables and independent *t*-tests or one-way ANOVA for continuous variables, as appropriate. Multiple linear regression analysis was conducted to identify factors associated with fertility preservation behaviours. The behaviour score served as the dependent variable, and demographic characteristics and HBM components were entered as independent variables using stepwise selection with entry criterion *p* < 0.10 and removal criterion *p* > 0.15. Statistical significance was set at *p* < 0.05, and all tests were two-tailed.

## 3 Results

### 3.1 Demographic characteristics

Among the 294 participants, the majority were urban residents (93.54%) with an average age of childbearing years. It showed a highly educated sample (80.61% college-educated), with healthcare workers representing the largest occupational group (45.24%), providing a unique perspective on a population with presumably greater health literacy (see Table 1 for details).

**Table 1:** Demographic characteristics of study participants (*N* = 294).

Characteristics	*n*	%
Age (years)		
18–25	42	14.29
26–35	168	57.14
36–49	84	28.57
Residence		
Urban	275	93.54
Rural	8	2.72
Suburban	11	3.74
Education Level		
Primary school and below	1	0.34
Middle school	13	4.42
High school/Technical school	16	5.44
College/University	237	80.61
Graduate and above	27	9.18
Marital Status		
Single	48	16.33
Married	238	80.95
Divorced	8	2.72
Widowed	0	0
Number of Children		
No children	70	23.81
One child	158	53.74
Two children	65	22.11
Three or more	1	0.34
Occupation		
Healthcare workers	133	45.24
Civil servants	63	21.43
Education workers	22	7.48
Workers/Farmers	19	6.46
Students	17	5.78
Others	40	13.61
Economic Status		
Very good	4	1.36
Good	39	13.27
Average	235	79.93
Poor	12	4.08
Very poor	4	1.36
Medical insurance coverage		
New Rural Cooperative Medical Scheme	7	2.38
Urban Resident Basic Medical Insurance	45	15.31
Urban Employee Basic Medical Insurance	225	76.53
Government-sponsored Free Medical Care	6	2.04
Commercial Health Insurance	2	0.68
No Medical Insurance Coverage	9	3.06

### 3.2 Fertility preservation

Although 62.93% reported fertility awareness, only 31.97% underwent regular fertility assessments, suggesting awareness alone is insufficient to drive preventive healthcare seeking. In addition, high compliance with hygiene during sexual activity (85.03%) and substance avoidance (smoking 71.77%, alcohol 75.17%) indicates these are more readily adopted behaviours, whereas clinical engagement (assessments 31.97%, supplements 27.55%) represents a critical area for improvement. This dichotomy suggests that different intervention approaches may be needed for lifestyle versus clinical preventive behaviours.

### 3.3 Health belief model analysis

**Figure 1:**
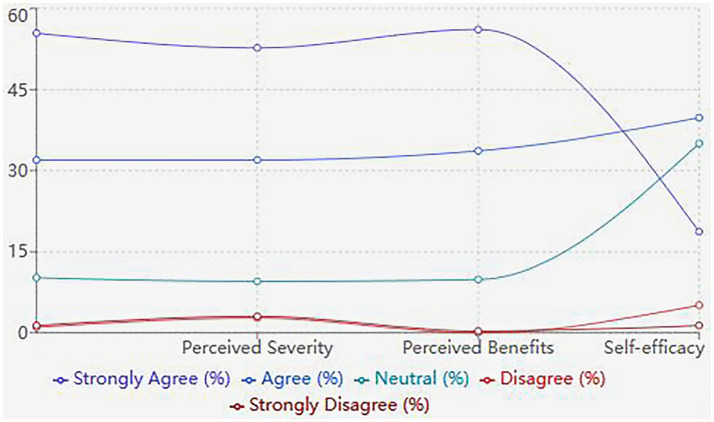
Patterns of health beliefs and fertility preservation behaviours among women of childbearing age.

As shown in Figure 1, although perceived susceptibility and benefits were high, this did not translate to action, challenging the assumptions of the HBM. The identified barriers – time constraints (50.34% found it difficult to allocate time for hospital visits), cost (25.17% considered the cost burdensome) and knowledge gaps (21.43% reported inadequate understanding of fertility preservation) – provide concrete targets for policy and education programmes. Notably, only 54.42% expressed confidence in maintaining lifestyle changes, revealing an underappreciated challenge in sustained behaviour modification.

### 3.4 Factors influencing fertility preservation behaviours

Multiple linear regression analysis revealed several significant predictors of fertility preservation behaviours (see Table 2). Education level (*β* = 0.326, *p* < 0.001), healthcare occupation (*β* = 0.284, *p* < 0.001) and perceived benefits (*β* = 0.253, *p* < 0.001) were positively associated with protective behaviours.

**Table 2:** Multiple linear regression analysis of factors influencing fertility preservation behaviours.

Variables	*β*	SE	*t*	*p*-value	95% CI
Education level	0.326	0.042	7.762	<0.001	0.243–0.409
Healthcare occupation	0.284	0.039	7.282	<0.001	0.207–0.361
Perceived benefits	0.253	0.037	6.837	<0.001	0.180–0.326
Age	−0.182	0.033	−5.515	<0.001	−0.247--0.117
Marital status	0.165	0.031	5.323	<0.001	0.104–0.226
Economic status	0.147	0.029	5.069	<0.001	0.090–0.204
Urban residence	0.138	0.028	4.929	<0.001	0.083–0.193
Self-efficacy	0.125	0.027	4.630	<0.001	0.072–0.178

*β* = standardised regression coefficient; SE = standard error; CI = confidence interval *R*^2^ = 0.563, Adjusted *R*^2^ = 0.551, *F* = 45.872, *p* < 0.001.

## 4 Discussion

Our study found a significant gap between fertility preservation awareness (62.93%) and practice (31.97%), with structural factors (education, occupation) influencing behaviours more than health beliefs, highlighting a need for targeted interventions.

The high prevalence of awareness regarding fertility-related concepts among participants reflects a growing consciousness about reproductive health. However, this level of awareness has not consistently translated into protective behaviours, particularly in areas requiring sustained lifestyle modifications or medical interventions. This knowledge–behaviour gap aligns with previous research indicating that awareness alone may be insufficient to drive behavioural change in reproductive health practices (14). Similar discrepancies have been documented in studies from other Asian countries, where cultural and social factors significantly influence health-seeking behaviours (15).

Behavioural adoption patterns showed significant differences, with high compliance in personal hygiene and lifestyle contrasting sharply with clinical fertility assessments. This suggests that participants may face potential barriers in accessing specialised healthcare services and are more likely to adopt behaviours that are easily integrated into daily routines and require minimal external resources (16, 17). Given the growing evidence that micronutrients have a protective effect on reproductive health (18), the particularly low acceptance rate of nutritional supplements is concerning, suggesting an opportunity for improved education and awareness in this area.

The HBM components revealed interesting patterns that merit careful consideration. The high levels of perceived susceptibility and severity (>87%) indicate that participants generally recognise the risks associated with inadequate fertility protection. This awareness level is notably higher than reported in previous studies (19), possibly reflecting improved health education and information accessibility. However, the disconnect between risk awareness and preventive behaviours suggests the influence of other factors beyond risk perception.

The identified barriers to fertility preservation, particularly time constraints and financial concerns, reflect systemic challenges in healthcare accessibility, highlighting the impact of socioeconomic factors on reproductive healthcare utilisation (20). Additionally, the lower confidence levels in maintaining long-term lifestyle changes suggest a need for sustained support systems and interventions that address behavioural maintenance rather than just initiation (21).

The significant association between education level and protective behaviours indicates that for each additional level of educational attainment, protective behaviours increase by nearly one-third of a standard deviation, supporting existing literature on the role of education in health outcomes (22). Healthcare workers exhibited significantly higher engagement in protective behaviours, suggesting that professional knowledge and familiarity with healthcare systems may facilitate better health practices (23). These professional advantages may partially explain the overall high educational attainment in our sample (80.61% with a college degree or higher); however, this also indicates potential selection bias, necessitating future studies with more diverse samples.

Demographic predictors align with established patterns; married women are more actively engaged in protective behaviours, supporting the importance of partner support in health decision-making (24), and age patterns reflect life stage considerations in reproductive planning (25). In our sample, the urban population predominated (93.54%), highlighting persistent regional disparities in healthcare access and resource distribution (26), although this limited the generalisability of the findings to rural populations.

## 5 Conclusion

This study reveals complex interactions between knowledge, beliefs and practices in fertility preservation among women of childbearing age. Although awareness of the importance of fertility preservation is generally high, significant barriers exist in translating this knowledge into sustained protective behaviours. The findings highlight the need for comprehensive interventions that address both individual and systemic barriers to fertility preservation. Healthcare providers and policymakers should consider these factors when developing strategies to promote fertility preservation among women of childbearing age. The development of accessible, sustainable and culturally appropriate interventions will be crucial in improving fertility preservation practices in this population.

## Data Availability

The raw data supporting the conclusions of this article will be made available by the authors, without undue reservation.
