# Supplementary material for: Knowledge, attitudes, and practices regarding fertility preservation among women of childbearing age in southern China: a cross-sectional study
Source: Front Public Health. 2025 Oct 3;13:1612784. doi: 10.3389/fpubh.2025.1612784 (PMC12531196; doi:10.3389/fpubh.2025.1612784)
Supplement: Supplementary file 1 [file Table_1.docx]

## Supplementary Table S1. Detailed Item List and Scoring Methods for Fertility Preservation Behaviors and Health Belief Model Constructs

| **Dimension/**  **Variable** | **Item Examples** | **Scoring Method** | **Summation Algorithm** | **Cronbach's α** |
| --- | --- | --- | --- | --- |
| **Fertility Preservation Behaviors** | | | | |
| Dietary Habits | • Daily fresh fruits/vegetables ≥400g | 1-5 Likert scale  (1=almost never to 5= very often) | Direct sum of 6 items | 0.84 |
|  | • Limiting high-sugar beverages |  |  |  |
|  | • Regular meal timing |  |  |  |
|  | • Avoiding processed foods |  |  |  |
|  | • Adequate water intake |  |  |  |
|  | • Balanced nutrition |  |  |  |
| Exercise Patterns | • ≥150 min/week moderate exercise | 1-5 Likert scale | Direct sum of 4 items | 0.81 |
|  | • Regular physical activity |  |  |  |
|  | • Avoiding sedentary behavior |  |  |  |
|  | • Strength training exercises |  |  |  |
| Lifestyle Choices | • Smoking avoidance | 1-5 Likert scale | Direct sum of 8 items | 0.86 |
|  | • Alcohol limitation |  |  |  |
|  | • Regular sleep patterns |  |  |  |
|  | • Stress management |  |  |  |
|  | • Weight control |  |  |  |
|  | • Avoiding environmental toxins |  |  |  |
|  | • Safe sexual practices |  |  |  |
|  | • Personal hygiene |  |  |  |
| Healthcare Behaviors | • Regular fertility assessments | 1-5 Likert scale | Direct sum of 6 items | 0.83 |
|  | • Folic acid supplementation |  |  |  |
|  | • Routine gynecological check-ups |  |  |  |
|  | • Vaccination compliance |  |  |  |
|  | • Seeking professional advice |  |  |  |
|  | • Medication adherence |  |  |  |
| **Total Behavior Score** | **Sum of all 24 items (Range: 24-120)** | | | **0.89** |
| **Health Belief Model Constructs** | | | | |
| Perceived Susceptibility | • Risk of fertility problems | 1-5 Likert scale(1=strongly disagreeto 5=strongly agree) | Direct sum of 6 items | 0.87 |
|  | • Likelihood of reproductive damage |  |  |  |
|  | • Vulnerability to infertility |  |  |  |
|  | • Age-related fertility decline |  |  |  |
|  | • Environmental risk exposure |  |  |  |
|  | • Genetic predisposition |  |  |  |
| Perceived Severity | • Impact on quality of life | 1-5 Likert scale | Direct sum of 6 items(Range: 6-30) | 0.85 |
|  | • Pregnancy complications |  |  |  |
|  | • Emotional consequences |  |  |  |
|  | • Financial burden |  |  |  |
|  | • Relationship effects |  |  |  |
|  | • Long-term health impact |  |  |  |
| Perceived Benefits | • Increased pregnancy succes | 1-5 Likert scale | Direct sum of 6 items | 0.90 |
|  | s• Reduced complications |  |  |  |
|  | • Better maternal health |  |  |  |
|  | • Improved fetal outcomes |  |  |  |
|  | • Peace of mind |  |  |  |
|  | • Cost-effectiveness |  |  |  |
| Perceived Barriers | • Time constraints | 1-5 Likert scale* | Direct sum of 8 items | 0.82 |
|  | • Financial limitations |  |  |  |
|  | • Lack of knowledge |  |  |  |
|  | • Healthcare access |  |  |  |
|  | • Social stigma |  |  |  |
|  | • Partner resistance |  |  |  |
|  | • Work conflicts |  |  |  |
|  | • Geographic distance |  |  |  |
| Self-efficacy | • Confidence in lifestyle changes | 1-5 Likert scale | Direct sum of 5 items | 0.88 |
|  | • Ability to seek medical help |  |  |  |
|  | • Overcoming barriers |  |  |  |
|  | • Long-term maintenance |  |  |  |
|  | • Decision-making skills |  |  |  |

*Note: Negatively worded items in the Perceived Barriers subscale were reverse-coded before summation. All HBM construct scores were standardized using z-score transformation for regression analysis.
